# Influences of genetic factors for educational attainment and cognitive functions on current dietary consumption

**DOI:** 10.1016/j.isci.2025.113530

**Published:** 2025-09-08

**Authors:** Daisuke Fujikane, Kazutaka Ohi, Daisuke Nishizawa, Junko Hasegawa, Naomi Sato, Fumihiko Tanioka, Haruhiko Sugimura, Kazutaka Ikeda, Toshiki Shioiri

**Affiliations:** 1 Department of Psychiatry, Gifu University Graduate School of Medicine, Gifu, Japan; 2 Addictive Substance Project, Tokyo Metropolitan Institute of Medical Science, Tokyo, Japan; 3 Department of Neuropsychopharmacology, National Institute of Mental Health, National Center of Neurology and Psychiatry (NCNP), Tokyo, Japan; 4 Department of Clinical Nursing, Hamamatsu University School of Medicine, Shizuoka, Japan; 5 Department of Pathology, Iwata City Hospital, Shizuoka, Japan; 6 Sasaki Institute Sasaki Foundation, Tokyo, Japan

**Keywords:** Health sciences, Medicine, Medical specialty, Clinical genetics, Geriatrics, Public health

## Abstract

Dietary habits are critical for the prevention and management of physical and mental disorders and may also be influenced by genetic factors for educational attainment (EA) and cognitions. This study examined the effects of polygenic scores (PGSs) associated with EA and cognitions on current dietary consumption among older patients with lifestyle-related diseases. Dietary habits were assessed across eight categories in 730 older adult outpatients diagnosed with or suspected of having lifestyle-related diseases. Six PGSs associated with EA and cognitions were calculated using large-scale genome-wide association study (GWAS) datasets, and their correlations with dietary consumption patterns were investigated. Higher PGSs associated with EA and cognitions were commonly correlated with increased consumption of fruits. Moreover, we found positive genetic correlations between fruit consumption with EA and cognitive functions in an independent cohort. Our findings suggest that genetic factors related to educational and cognitive phenotypes may shape dietary habits that promote physical and mental health and longevity.

## Highlights

• Polygenic scores (PGSs) related to EA and cognition were tested for links to current diet
• Higher EA and cognitive PGSs were linked to more fruit intake in older adults
• UKBB data showed positive genetic links of fruit intake with EA and cognition
• Greater fruit intake may be affected by genetic factors related to EA and cognitions

## Graphical abstract

**Figure:**
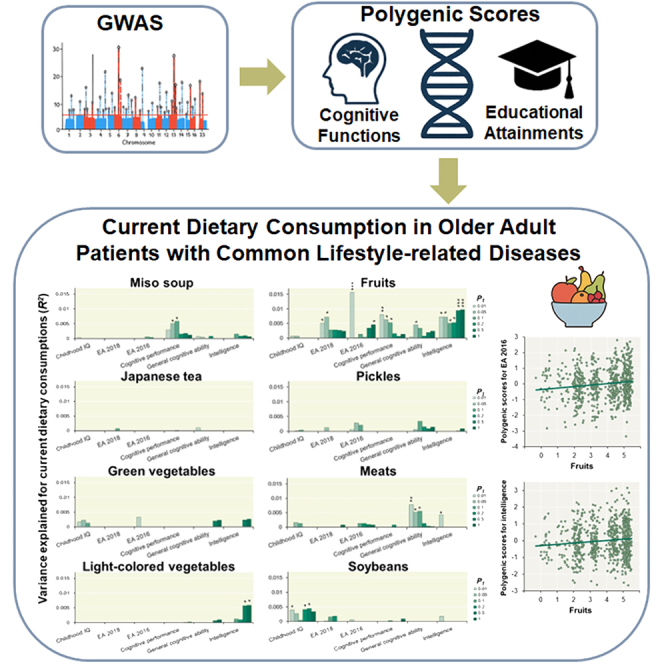

## Introduction

Diet is one of the most extensively studied aspects of human behavior, given its crucial impact on human well-being. The quality, quantity, and patterns of food consumption are associated with a broad spectrum of medical conditions, including metabolic, inflammatory, or mental disorders, as well as being influenced by environmental factors, such as socioeconomic status (SES).^1^^,^^2^^,^^3^ The complex interaction between genetic factors and lifestyle choices, notably dietary habits, has captured the attention of contemporary research in genetics and nutritional science.^4^^,^^5^ These studies are founded on the principle that although environmental factors significantly shape dietary habits, an individual’s genetic composition also plays a critical role in these choices.^6^ The development of polygenic scores has become a key instrument in elucidating how a multitude of genetic factors can cumulatively influence a wide range of human behaviors and factors, including educational attainment (EA), cognitive abilities, and even dietary preferences.^7^ While traditional genome-wide association studies (GWASs) focus on identifying genetic variants associated with a single phenotype, polygenic score analysis enables the examination of whether the genetic predisposition for one trait (e.g., EA or cognitive functions) is associated with a related but distinct outcome, such as dietary habits.^8^

EA and cognitive functions are complex traits influenced by the relationships among numerous genetic and environmental factors, with heritability estimates of approximately 40% for EA and 50% for cognitive functions.^9^^,^^10^ Findings from previous large-scale GWASs (*n* = 12,441–1,131,881) focusing on childhood intelligence quotient (IQ),^11^ EA,^12^^,^^13^ cognitive performance,^13^ general cognitive ability,^14^ and intelligence^15^ have revealed multiple genetic variants linked to education and cognition, highlighting the polygenic nature of these traits. EA and cognitive functions are crucial determinants of both physical and mental health outcomes.^16^^,^^17^^,^^18^ Additionally, both EA and cognitive functions are genetically correlated with the risk of physical diseases and several mental disorders.^19^ Similarly, dietary habits have been identified as key determinants of physical and mental health outcomes,^20^^,^^21^ particularly in elderly individuals, where nutrition plays a critical role in managing and preventing these conditions. However, the influence of genetic factors related to educational and cognitive phenotypes on dietary choices has been relatively underexplored.^20^ Given the observed associations of EA and cognitive functions and dietary habits with physical diseases and several mental disorders and considering the genetic influences of EA and cognitive functions, we hypothesized that polygenic scores associated with EA and cognitive functions would influence specific dietary behaviors, such as increased fruit and vegetable consumption and decreased intake of meats.

In this study, we focused on a cohort of 730 older adult outpatients with common lifestyle-related diseases (Table 1). By not limiting our analysis exclusively to individuals with specific physical diseases and mental disorders or healthy individuals, we aimed to ensure the generalizability of our findings across a broader spectrum of the older population. We investigated the effects of polygenic scores associated with EA and cognitive functions based on six large-scale GWAS datasets on the current consumption of eight specific dietary items in older patients. To capture cognitive-related genetic influences across the life span, we included polygenic scores derived from GWASs of both childhood and adult cognitive traits and examined their respective associations with current dietary consumption.

**Table 1:** Demographic information of 730 older adult participants with any lifestyle-related diseases

Variable	Participants (*n* = 730)
Age (years)	72.6 ± 6.5 (60–93)
Sex (male/female)	529/201
Height (cm)	158.7 ± 8.8 (130–185)
Weight (kg)	57.0 ± 10.0 (30–101)
Body mass index	22.6 ± 3.2 (13.8–35.4)
Smoker (current/past/never)	141/360/229
Alcohol drinker (current/past/never)	303/108/318
Current cancer (yes/no/unknown)	71/633/26
Current diabetes mellitus (yes/suspected/no/unknown)	128/16/571/15
Current hypertension (yes/no/unknown)	282/422/26
Past cancer (yes/no/unknown)	99/623/8
Past diabetes mellitus (yes/suspected/no/unknown)	8/14/700/8
Past hypertension (yes/no/unknown)	41/680/9

**Current dietary habits**	

Miso soup (0–5 scale)	4.3 ± 1.3 (0–5)
Japanese tea (0–5 scale)	4.8 ± 0.8 (0–5)
Green and yellow vegetables (0–5 scale)	4.5 ± 0.9 (1–5)
Light-colored vegetables (0–5 scale)	4.6 ± 0.9 (0–5)
Fruits (0–5 scale)	3.8 ± 1.5 (0–5)
Pickles (0–5 scale)	3.4 ± 1.9 (0–5)
Meats (0–5 scale)	2.4 ± 1.1 (0–5)
Soybeans (0–5 scale)	4.3 ± 1.1 (0–5)

Complete demographic information was not obtained for all the participants (height, *n* = 729; weight, *n* = 727; body mass index, *n* = 726; alcohol drinker, *n* = 729; light-colored vegetables, *n* = 729; fruits, *n* = 727; pickles, *n* = 728; meats, *n* = 729). The means ± SD (range) are shown.

## Results

### Effects of polygenic scores associated with EA and cognitive functions on eight types of current dietary consumption

**Figure 1:**
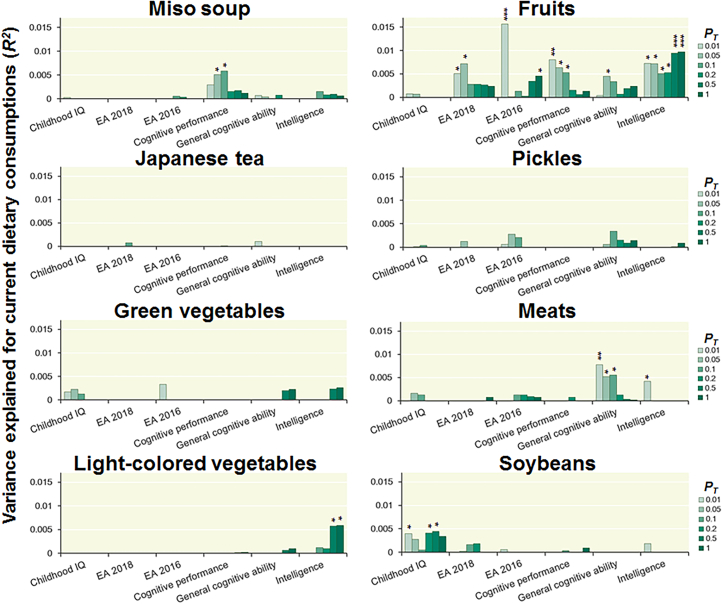
Effects of polygenic scores associated with childhood IQ, EA, cognitive performance, general cognitive ability, and intelligence at different *P*_*T*_ levels on eight current dietary consumptions in older patients with any lifestyle-related diseases EA, educational attainment. ^∗^*p* < 0.05, ^∗∗^*p* < 0.01, ^∗∗∗^*p* < 6.25 × 10^−3^.

**Figure 2:**
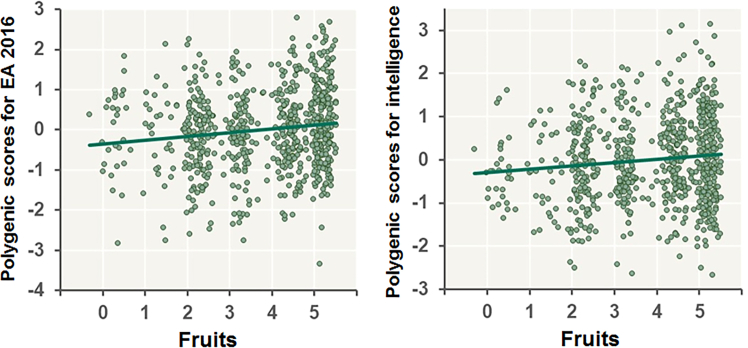
Correlations between polygenic scores associated with EA and intelligence and the frequency of fruit consumption The polygenic scores corrected for the cohort were *Z* standardized. Dietary consumption was corrected for age and sex. EA, educational attainment.

We investigated the impacts of six polygenic scores associated with EA and cognitive functions, childhood IQ, EA2016, EA2018, cognitive performance, general cognitive ability, and intelligence, on the frequencies of eight specific types of dietary consumption at varying *P*_*T*_ levels (Figure 1 and Table S3). Notably, fruit consumption frequency was commonly influenced by polygenic scores associated with EA2018 (maximum at *P*_*T*_ < 0.05: *R*^*2*^ = 0.0072, *p* = 0.010), EA2016 (maximum at *P*_*T*_ < 0.01: *R*^*2*^ = 0.0156, *p* = 2.89 × 10^−4^), cognitive performance (maximum at *P*_*T*_ < 0.01: *R*^*2*^ = 0.0080, *p* = 7.31 × 10^−3^), general cognitive ability (maximum at *P*_*T*_ < 0.05: *R*^*2*^ = 0.0045, *p* = 0.034), and intelligence (maximum at *P*_*T*_ ≤ 1: *R*^*2*^ = 0.0097, *p* = 3.49 × 10^−3^), except for polygenic scores associated with childhood IQ (*p* > 0.05). Higher polygenic scores associated with EA and intelligence significantly correlated with increased fruit consumption (Figure 2). Even after adjusting for current disease status (cancer, hypertension, or diabetes mellitus), the associations between polygenic scores related to EA and intelligence and fruit consumption remained significant (Table S4).

Furthermore, polygenic scores associated with childhood IQ were marginally positively correlated with soybean intake frequency (maximum at *P*_*T*_ < 0.5: *R*^*2*^ = 0.0044, *p* = 0.037), and polygenic scores associated with cognitive performance were marginally positively correlated with miso soup consumption frequency (maximum at *P*_*T*_ < 0.1: *R*^*2*^ = 0.0059, *p* = 0.022). The polygenic scores associated with intelligence were marginally positively correlated with light-colored vegetable consumption frequency (maximum at *P*_*T*_ ≤ 1: *R*^*2*^ = 0.0059, *p* = 0.018). Conversely, polygenic scores associated with general cognitive ability and intelligence showed marginally negative correlations with meat intake frequency (general cognitive ability, maximum at *P*_*T*_ < 0.01: *R*^*2*^ = 0.0078, *p* = 9.61 × 10^−3^; intelligence, maximum at *P*_*T*_ < 0.01: *R*^*2*^ = 0.0042, *p* = 0.044). No significant correlations were detected between these polygenic scores and the intake frequencies of Japanese tea, green vegetables, or pickles (*p* > 0.05).

### Genetic correlations between fruit consumption and EA and cognitive functions

**Figure 3:**
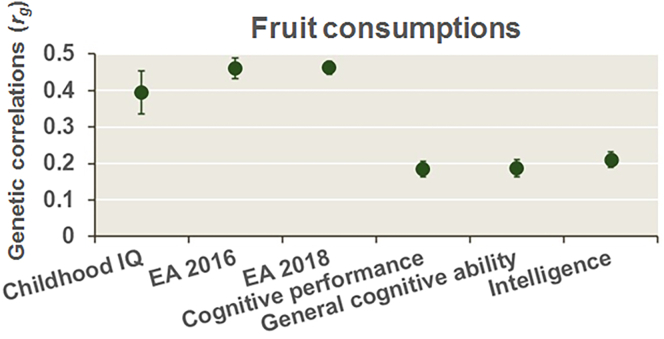
Genetic correlations (*r*_*g*_) of fruit consumption with EA and cognitive functions in the general population A positive *r*_*g*_ indicates that higher consumption of fruits was genetically correlated with higher EA and cognitive functions. The error bars represent the standard error.

Based on the impacts of polygenic scores associated with EA and cognitive functions on fruit consumption in our participants, we further explored genetic correlations between fruit consumption from GWASs in independent UK Biobank (UKBB) participants (https://www.ebi.ac.uk/gwas/)^20^ and six GWASs of educational and cognitive phenotypes using linkage disequilibrium score regression analyses (Figure 3). All the educational and cognitive phenotypes were genetically positively correlated with fruit consumption (Figure 3; *r*_*g*_ values ranging from 0.18 for cognitive performance to 0.46 for EA 2016 and EA 2018; all *p* < 6.25 × 10^−3^).

## Discussion

This study is the first to investigate whether polygenic scores associated with EA and cognitive functions influence current specific dietary consumption in older patients diagnosed with or suspected of having lifestyle-related diseases. Higher polygenic scores associated with EA and cognitive functions, with the exception of childhood IQ, are commonly associated with increased fruit consumption in older patients. Among other types of dietary consumption, higher intakes of soybeans, miso soup, and light-colored vegetables and lower meat intake frequencies were marginally influenced by polygenic scores associated with cognitive functions. Furthermore, positive genetic correlations of fruit consumption in independent UKBB individuals with all six educational and cognitive phenotypes were revealed. These findings indicate that greater fruit intake may be affected by genetic factors related to EA and cognitive functions, such as physical and mental disorders,^19^ in older patients with lifestyle-related diseases as well as in the general population.

Generally, consuming fruits offers numerous health benefits. As they are rich in vitamins, minerals, dietary fiber, and antioxidants, these nutrients support immune function, reduce the risk of several diseases, enhance digestive health, and may play a role in alleviating mental disorders, thus contributing to overall well-being.^22^^,^^23^^,^^24^^,^^25^^,^^26^ Consequently, individuals with a genetic predisposition associated with EA and cognitive functions may adopt behaviors that lead to increased fruit intake for their overall health and mental well-being via high EA and cognitive functions. This tendency not only highlights the potential role of genetic factors for EA and cognitive functions in shaping dietary habits that promote physical and mental health and longevity but also suggests that such dietary preferences might be associated with a lower risk of physical and mental disorders.

In older patients who were diagnosed with or suspected of having lifestyle-related diseases, such as cancer, diabetes mellitus, and hypertension, we observed positive correlations between fruit consumption and polygenic scores associated with EA and cognitive function. These current dietary habits may have been established earlier in life, while their current dietary habits might have been modified upon the diagnosis of lifestyle-related diseases. Among the eight dietary items examined, healthier dietary patterns were noted, including higher intakes of Japanese tea, green and yellow vegetables, light-colored vegetables, and soybeans and lower meat consumption. Thus, if dietary habits change after the onset of lifestyle-related diseases, dietary items other than fruits could also be influenced by these polygenic scores. Considering the genetic correlations between fruit consumption in independent UKBB participants and educational and cognitive phenotypes, our findings suggest that dietary habits related to fruit intake are more sensitive to genetic factors associated with EA and cognitive functions throughout life, at least before the onset of lifestyle-related diseases.

For the prevention and management of patients with lifestyle-related diseases, such as cancer, diabetes mellitus, and hypertension, fruit intake can offer several benefits, including essential vitamins, minerals, antioxidants, and dietary fiber. These nutrients support overall health, aid in disease prevention and management, and potentially reduce disease risk factors.^27^^,^^28^ However, fruit intake must be carefully managed, especially in individuals with diabetes, due to its natural sugar content, which can impact blood glucose levels.^29^ Additionally, for individuals with certain cancers or who are receiving specific treatments, high fiber intake from fruits may cause digestive discomfort.^30^ Therefore, while fruits are recommended as part of dietary therapy for these diseases, their consumption should be personalized to individual nutritional needs and health conditions.

Bidirectional causal associations between EA and fruit consumption have been revealed.^20^ A relatively high EA significantly led to increased fruit intake, whereas relatively high fruit consumption, conversely, nominally led to increased EA.^20^ These causal associations between EA and food intake are complex, as EA is partially positively correlated with SES, i.e., higher incomes or occupational choice.^16^^,^^31^ Therefore, SES might impact the associations between EA and fruit consumption. In contrast, few studies have investigated possible causal associations between cognitive functions and fruit consumption. Future investigations should address how the bidirectional causal associations between EA and cognitive functions and fruit consumption change when SES is considered.

In conclusion, we investigated the complex relationships between genetic factors for educational and cognitive phenotypes and current dietary habits among older adults with lifestyle-related diseases. We revealed significant influences of polygenic scores associated with EA and cognitive functions on specific dietary behaviors, notably increased consumption of fruits, in patients and positive genetic correlations of fruit intake with EA and cognitive functions in independent individuals in the general population, suggesting that genetic factors related to educational and cognitive phenotypes influence dietary choices throughout life. Our findings may contribute to the growing field of nutritional genetics and highlight the potential for personalized dietary recommendations based on genetic factors to prevent and manage physical and mental disorders.

### Limitations of the study

There are several limitations in the interpretation of our findings. We did not detect significant correlations between polygenic scores associated with childhood IQ and fruit consumption in our participants. The efficacy of polygenic score analysis depends on the sample size of the discovery GWAS; therefore, the relatively small sample size of the GWAS for childhood IQ (Table 2) might have affected our results. Our questionnaire does not comprehensively cover all dietary habits, as it does not include some items, such as grains, rice, fish, eggs, milk, coffee, and snacks. While we have accounted for genetic factors and dietary behaviors, other confounding factors, such as SES, lifestyle choices, physical activity levels, and unmeasured environmental influences, may also play important roles in these associations but were not fully explored in the current study. Our cohort, comprising older adult outpatients with lifestyle-related diseases, may not accurately represent the general population, potentially limiting the generalizability of our results. We did not directly assess EA or cognitive functions in our participants. In particular, direct assessments of EA, cognitive performance (e.g., IQ tests), and functional outcomes (e.g., activities of daily living) were not available, preventing validation of polygenic scores against actual cognitive or behavioral phenotypes in this cohort. Future research should aim to overcome these limitations by incorporating longitudinal study designs, a broader range of confounding variables, and more diverse populations to better elucidate the complex relationships between genetic factors for human behaviors and dietary habits.

**Table 2:** Descriptive information for six GWASs of EA and cognitive functions for polygenic score calculations

		PMID	GWAS significant loci	*N*
Childhood IQ	Benyamin et al.^11^	23358156	0	12,441
EA 2016	Davies et al.^12^	27046643	15	111,114
EA 2018	Lee et al.^13^	30038396	1,271	1,131,881
Cognitive performance	Lee et al.^13^	30038396	225	257,841
General cognitive ability	Davies et al.^14^	29844566	148	282,014
Intelligence	Savage et al.^15^	29942086	205	269,867

## Resource availability

### Lead contact

Further information and requests for resources should be directed to and will be fulfilled by the lead contact, Kazutaka Ohi (ohi.kazutaka.h8@f.gifu-u.ac.jp).

### Materials availability

No new materials were generated in this study.

### Data and code availability

• The data are not publicly available because they contain information that could compromise research participant privacy/consent.
• No custom code was generated for this study.
• Additional information may be provided by the lead contact within the limits of ethical and legal considerations.

## Acknowledgments

This work was supported by Grants-in-Aid for Scientific Research (C) (22K07614, 25K10832, and 25K10808) and 10.13039/501100001691KAKENHI Advanced Animal Model Support (AdAMS) (16H06276 and AC220024) from the 10.13039/501100001691Japan Society for the Promotion of Science (10.13039/501100001691JSPS), 10.13039/100009619AMED under grant number JP21uk1024002, a grant from the 10.13039/501100005865Mochida Memorial Foundation for Medical and Pharmaceutical Research, and a grant from the 10.13039/100007449Takeda Science Foundation. We would like to thank all of the individuals who participated in this study.

## Author contributions

K.O. supervised the entire project. K.O. and D.F. wrote the manuscript and were critically involved in the design, analysis, and interpretation of the data. K.O., D.F., and T.S. were responsible for performing the literature review. D.N., J.H., N.S., F.T., H.S., and K.I. were heavily involved in the collection of the majority of the data and intellectually contributed to data interpretation. All authors contributed to and have approved the final manuscript.

## Declaration of interests

The authors declare no competing interests.

## STAR★Methods

### Key resources table

**Table:** 

REAGENT or RESOURCE	SOURCE	IDENTIFIER
**Biological samples**		

Human peripheral blood samples (for genotyping)	This paper	N/A

**Critical commercial assays**		

HumanCytoSNP v2.0 BeadChip	Illumina	Cat#WG-320-2101
HumanCoreExome v1.0 BeadChip	Illumina	Cat#WG-331-1001

**Deposited data**		

GWAS summary statistics for EA and cognitive functions	Lee et al.,^13^ Savage et al.,^15^ Davies et al.,^12^^,^^14^ Benyamin et al.^11^	See ref.^11^^,^^12^^,^^13^^,^^14^^,^^15^

**Software and algorithms**		

PLINK v1.9	Purcell et al.^7^	https://www.cog-genomics.org/plink/
IBM SPSS Statistics 28.0	IBM Japan	https://www.ibm.com/products/spss-statistics
LDSC (LD Score Regression)	Bulik-Sullivan et al.^32^	https://github.com/bulik/ldsc

### Experimental model and study participant details

#### Target participants

A total of 730 older adult outpatients aged 60 years and above were recruited at the Department of Clinical Laboratories, Iwata City Hospital, Shizuoka, Japan. These individuals visited for blood sampling during the recruitment period from 2003 to 2008^33^^,^^34^^,^^35^^,^^36^ (Table 1). The participants were primarily diagnosed with or suspected of having lifestyle-related diseases, such as cancer, diabetes mellitus, and hypertension. All the participants were unrelated, genetically homogeneous Japanese individuals, predominantly residing in the Tokai region of Japan. The eligibility criteria included being over 60 years of age, ambulatory, and capable of verbal communication. We did not assess current or past contact with psychiatric services or the use of psychiatric medication at recruitment. Written informed consent was obtained from all subjects. We assert that all procedures contributing to this work comply with the ethical standards of the relevant national and institutional committees on human experimentation and with the Helsinki Declaration of 1975, as revised in 2008. All procedures involving human subjects/patients were approved by the Institutional Review Boards of Iwata City Hospital and Hamamatsu University School of Medicine (21-8), Gifu University (2019-233), and Tokyo Institute of Psychiatry (now known as Tokyo Metropolitan Institute of Medical Science) (20-23(1)).

### Method details

#### Current specific dietary consumption

The eight specific types of dietary consumption were evaluated using a questionnaire.^33^^,^^34^ The questionnaire covered eight dietary items, including (i) miso soup, (ii) Japanese tea, (iii) green and yellow vegetables, (iv) light-colored vegetables, (v) fruits, (vi) pickles, (vii) meats, and (viii) soybeans. Responses were recorded on a 6-point scale: 0 (rarely); 1 (1–3 days a month); 2 (1–2 days a week); 3 (3–4 days a week); 4 (5–6 days a week); and 5 (daily) consumption. The correlations among these dietary habits were relatively weak (*r*<0.35), except for a moderate correlation between green and yellow vegetable consumption and light-colored vegetable consumption (*r*=0.63).^36^ Furthermore, to explore the potential influence of clinical status on dietary behaviors, we examined differences in dietary consumption between participants with and without current diagnoses of cancer, hypertension, and diabetes mellitus (Table S1). No significant differences in dietary habits were observed between groups, except for increased intake of green and yellow vegetables among participants with cancer (*p*=0.022) and reduced consumption of miso soup (*p*=0.042) and soybeans (*p*=0.0022) among those with hypertension, suggesting that current disease status may not have a substantial impact on overall dietary behaviors in our cohort.

#### Genotyping and quality control

A comprehensive overview of the genotyping and quality control (QC) methods used in the study has been provided previously.^35^ Briefly, peripheral venous blood samples were collected for DNA extraction. The participants were genotyped using two types of whole-genome genotyping arrays: the HumanCytoSNP v2.0 BeadChip (*n*=300) or the HumanCoreExome v1.0 BeadChip (*n*=430) (Illumina, San Diego, CA, USA). During the QC process, samples with a genotype call rate of less than 0.95 and single-nucleotide polymorphisms (SNPs) with a genotype call frequency of less than 0.95 or a ‘cluster sep’ (an index of genotype cluster separation) of less than 0.1 were excluded.^35^ After this process, a total of 225,602 SNPs for HumanCytoSNP and 256,997 SNPs for HumanCoreExome were retained.^35^^,^^36^

#### Polygenic score calculations

The polygenic scores for our target patients were calculated from six large-scale GWASs, focusing on childhood IQ,^11^ EA2016,^12^ EA2018,^13^ cognitive performance,^13^ general cognitive ability,^14^ and intelligence.^15^ To identify relevant SNPs, along with their *p* values and effect sizes (*beta*) related to EA and cognitive functions, we utilized six publicly available GWAS datasets as discovery samples.^12^^,^^13^^,^^14^^,^^15^ SNPs in linkage disequilibrium (LD) within our target patients were pruned using PLINK v1.9, applying a pairwise *r*^*2*^ threshold of 0.25 and a window size of 200 SNPs. Following this pruning process and the exclusion of SNPs located on sex and mitochondrial chromosomes, 72,223 independent SNPs for HumanCytoSNP and 68,313 independent SNPs for HumanCoreExome were retained. We calculated polygenic scores associated with each EA and cognitive function at varying levels of significance within the discovery GWAS datasets, with the following *P*_*T*__*cutoff*_ values: *P*_*T*_<0.01, *P*_*T*_<0.05, *P*_*T*_<0.1, *P*_*T*_<0.2, *P*_*T*_<0.5, and *P*_*T*_≤1. For each target patient, the polygenic score was computed by summing the product of the relevant alleles (0, 1, or 2) multiplied by the effect size across all SNPs in the *P*_T_-SNP sets. The number of SNPs used for each phenotype at each *P*_*T*_ threshold, stratified by genotyping array, is shown in Table S2.

### Quantification and statistical analysis

Statistical analyses were performed using IBM SPSS Statistics 28.0 software (IBM Japan, Tokyo, Japan). To assess the correlations between the polygenic scores related to educational and cognitive phenotypes at each *P*_*T*_ and the current dietary consumption of our patients, linear regression was used with current dietary intake as the dependent variable; polygenic scores related to educational and cognitive phenotypes as the independent variables; and age, sex, and array type as covariates. The adjusted *R*^*2*^ value indicates the proportion of variance in current dietary consumption explained by the polygenic scores. To isolate the variance specifically attributable to the polygenic scores, we subtracted the adjusted *R*^*2*^ value for the covariates alone (age, sex, and array type) from that of the full models. Genetic SNP correlations (*r*_*g*_) from GWASs were estimated using LDSC analysis.^32^^,^^37^^,^^38^^,^^39^^,^^40^^,^^41^^,^^42^ The nominal significance level was set at *p* < 0.05. Although polygenic scores at each *P*_*T*_ were highly correlated and not independent, *p* values derived from different *P*_*T*_ values were not adjusted for multiple comparisons. To mitigate type I errors, we applied a Bonferroni correction, setting a *p* value threshold of *p* < 6.25×10^-3^ (*α*=0.05/eight current dietary consumption).
