# Supplementary material for: Influences of genetic factors for educational attainment and cognitive functions on current dietary consumption
Source: iScience. 2025 Sep 8;28(10):113530. doi: 10.1016/j.isci.2025.113530 (PMC12493237; doi:10.1016/j.isci.2025.113530)
Supplement: Document S1. Tables S1–S4 [file mmc1.pdf]

iScience, Volume *28*

Supplemental information

Influences of genetic factors for educational attainment and cognitive functions on current dietary consumption

Daisuke Fujikane, Kazutaka Ohi, Daisuke Nishizawa, Junko Hasegawa, Naomi Sato, Fumihiko Tanioka, Haruhiko Sugimura, Kazutaka Ikeda, and Toshiki Shioiri

Supplementary Table 1. Differences in current dietary habits between participants with and without lifestyle-related diseases (Cancer, Diabetes mellitus, and Hypertension)

	Current cancer			Current diabetes mellitus				Current hypertension		
	Yes (n=71)	No (n=633)	p (β)	Yes (n=128)	Suspected (n=16)	No (n=571)	p (β)	Yes (n=282)	No (n=422)	p (β)
**Current dietary habits** (0-5 scale)										
Miso soup	4.4 ± 1.2	4.3 ± 1.3	0.44 (-0.03)	4.3 ± 1.3	3.6 ± 1.5	4.3 ± 1.2	0.40 (0.03)	4.2 ± 1.4	4.4 ± 1.2	**0.042 (0.08)**
Japanese tea	4.8 ± 0.9	4.8 ± 0.8	0.87 (0.01)	4.7 ± 1.1	5.0 ± 0.0	4.9 ± 0.8	0.09 (0.06)	4.8 ± 0.9	4.8 ± 0.8	0.66 (0.02)
Green and yellow vegetables	4.7 ± 0.7	4.5 ± 1.0	**0.022 (-0.08)**	4.7 ± 0.8	4.8 ± 0.8	4.5 ± 1.0	0.16 (-0.05)	4.5 ± 0.9	4.5 ± 0.9	0.63 (0.02)
Light-colored vegetables	4.7 ± 0.8	4.5 ± 0.9	0.09 (-0.06)	4.7 ± 0.7	4.8 ± 0.8	4.5 ± 0.9	0.09 (-0.06)	4.6 ± 0.9	4.5 ± 0.9	0.81 (-0.01)
Fruits	4.0 ± 1.3	3.8 ± 1.5	0.09 (-0.06)	4.0 ± 1.4	4.3 ± 1.1	3.8 ± 1.5	0.05 (-0.07)	3.7 ± 1.5	3.9 ± 1.4	0.14 (0.05)
Pickles	3.5 ± 2.1	3.4 ± 1.9	0.79 (-0.01)	3.3 ± 2.0	4.4 ± 1.1	3.4 ± 1.9	0.95 (0.00)	3.4 ± 2.0	3.4 ± 1.9	0.98 (0.00)
Meats	2.5 ± 1.4	2.4 ± 1.1	0.28 (-0.04)	2.3 ± 1.0	2.5 ± 1.1	2.4 ± 1.1	0.21 (0.05)	2.3 ± 1.1	2.4 ± 1.2	0.12 (0.06)
Soybeans	4.2 ± 1.1	4.3 ± 1.1	0.74 (0.01)	4.3 ± 1.1	4.6 ± 0.9	4.3 ± 1.1	0.78 (-0.01)	4.1 ± 1.3	4.4 ± 1.0	**0.0022 (0.11)**

Mean dietary intake frequencies ± SD on a 0–5 scale are presented for each group. To examine differences in current dietary habits between groups, linear regression analyses were performed with each dietary habit as the dependent variable, current disease status as the independent variable, and age, sex, and array type as covariates. P-values <0.05 are shown in boldface.

Supplementary Table 2. Number of SNPs included in each polygenic score associated with educational attainment (EA) and cognitive functions at different P_T thresholds across two genotyping arrays.

		HumanCytoSNP v2.0 (*n*=300)	HumanCoreExome v1.0 (*n*=430)
GWAS phenotype	P_T	Number of SNPs included	Number of SNPs included
Childhood IQ	0.01	474	389
	0.05	2,083	1,733
	0.1	4,112	3,486
	0.2	7,878	6,707
	0.5	18,732	15,875
	1	36,485	31,116
EA 2018	0.01	4,680	3,962
	0.05	9,642	8,299
	0.1	13,775	11,872
	0.2	20,296	17,583
	0.5	35,688	30,562
	1	57,662	49,306
EA 2016	0.01	1,353	1,204
	0.05	4,692	4,127
	0.1	8,209	7,084
	0.2	14,616	12,511
	0.5	31,740	27,069
	1	58,131	49,558
Cognitive performance	0.01	3,006	2,629
	0.05	7,442	6,511
	0.1	11,270	9,890
	0.2	17,753	15,520
	0.5	33,849	29,160
	1	57,663	49,307
General cognitive ability	0.01	2,572	2,232
	0.05	6,770	5,966
	0.1	10,687	9,273
	0.2	17,152	14,815
	0.5	33,537	28,874

	1	58,164	49,629
Intelligence	0.01	3,018	2,718
	0.05	7,498	6,652
	0.1	11,471	10,126
	0.2	18,042	15,637
	0.5	34,184	29,514
	1	57,993	49,718

Polygenic scores were calculated by summing the number of effect alleles (0, 1, or 2) at each SNP, weighted by the effect size estimated from the corresponding GWAS.

Supplementary Table 3. Adjusted R^2 values (p-values) indicating the proportion of variance in current dietary consumption explained by polygenic scores associated with childhood IQ, educational attainment (EA), cognitive performance (CP), general cognitive ability (g), and intelligence across different P_T thresholds.

Miso soup						
P_T	Childhood IQ	EA 2018	EA 2016	CP	g	Intelligence
0.01	<0.001 (0.27)	<0.001 (0.44)	<0.001 (0.61)	0.003 (0.08)	0.001 (0.22)	<0.001 (0.78)
0.05	<0.001 (0.68)	<0.001 (0.65)	<0.001 (0.40)	**0.005 (0.030)**	<0.001 (0.25)	<0.001 (0.38)
0.1	<0.001 (0.69)	<0.001 (0.54)	0.001 (0.23)	**0.006 (0.022)**	<0.001 (0.32)	0.002 (0.14)
0.2	<0.001 (0.48)	<0.001 (0.52)	<0.001 (0.26)	0.002 (0.15)	0.001 (0.21)	0.001 (0.20)
0.5	<0.001 (0.43)	<0.001 (0.62)	<0.001 (0.97)	0.002 (0.13)	<0.001 (0.39)	0.001 (0.19)
1	<0.001 (0.57)	<0.001 (0.79)	<0.001 (0.71)	0.001 (0.17)	<0.001 (0.38)	0.001 (0.23)
Japanese tea						
P_T	Childhood IQ	EA 2018	EA 2016	CP	g	Intelligence
0.01	<0.001 (0.94)	<0.001 (0.45)	<0.001 (0.35)	<0.001 (0.80)	0.001 (0.18)	<0.001 (0.77)
0.05	<0.001 (0.35)	<0.001 (0.40)	<0.001 (0.33)	<0.001 (0.96)	<0.001 (0.80)	<0.001 (0.84)
0.1	<0.001 (0.96)	0.001 (0.21)	<0.001 (0.33)	<0.001 (0.30)	<0.001 (0.51)	<0.001 (0.58)
0.2	<0.001 (0.85)	<0.001 (0.36)	<0.001 (0.42)	<0.001 (0.31)	<0.001 (0.86)	<0.001 (0.48)
0.5	<0.001 (0.88)	<0.001 (0.53)	<0.001 (0.90)	<0.001 (0.39)	<0.001 (0.64)	<0.001 (0.32)
1	<0.001 (0.59)	<0.001 (0.51)	<0.001 (0.97)	<0.001 (0.43)	<0.001 (0.69)	<0.001 (0.41)
Green vegetables						
P_T	Childhood IQ	EA 2018	EA 2016	CP	g	Intelligence
0.01	0.002 (0.12)	<0.001 (0.74)	0.003 (0.06)	<0.001 (0.99)	<0.001 (0.46)	<0.001 (0.70)
0.05	0.002 (0.09)	<0.001 (0.71)	<0.001 (0.33)	<0.001 (0.88)	<0.001 (0.64)	<0.001 (0.94)
0.1	0.001 (0.16)	<0.001 (0.30)	<0.001 (0.58)	<0.001 (0.85)	<0.001 (0.55)	<0.001 (0.59)
0.2	<0.001 (0.46)	<0.001 (0.31)	<0.001 (0.96)	<0.001 (0.89)	<0.001 (0.49)	<0.001 (0.37)
0.5	<0.001 (0.84)	<0.001 (0.56)	<0.001 (0.87)	<0.001 (0.57)	0.002 (0.11)	0.002 (0.09)
1	<0.001 (0.54)	<0.001 (0.44)	<0.001 (0.70)	<0.001 (0.53)	0.002 (0.09)	0.003 (0.08)
Light-colored vegetables						
P_T	Childhood IQ	EA 2018	EA 2016	CP	g	Intelligence
0.01	<0.001 (0.91)	<0.001 (0.65)	<0.001 (0.31)	<0.001 (0.82)	<0.001 (0.38)	<0.001 (0.85)
0.05	<0.001 (0.87)	<0.001 (0.75)	<0.001 (0.56)	<0.001 (0.98)	<0.001 (0.70)	<0.001 (0.46)
0.1	<0.001 (0.93)	<0.001 (0.52)	<0.001 (0.97)	<0.001 (0.75)	<0.001 (0.98)	0.001 (0.16)
0.2	<0.001 (0.89)	<0.001 (0.80)	<0.001 (0.95)	<0.001 (0.76)	<0.001 (0.82)	0.001 (0.18)
0.5	<0.001 (0.67)	<0.001 (0.93)	<0.001 (0.58)	<0.001 (0.29)	0.001 (0.22)	**0.006 (0.019)**

1	<0.001 (0.84)	<0.001 (0.81)	<0.001 (0.58)	<0.001 (0.28)	0.001 (0.18)	**0.006 (0.018)**
Fruits						
P_T	Childhood IQ	EA 2018	EA 2016	CP	*g*	Intelligence
0.01	0.001 (0.20)	**0.005 (0.027)**	**0.016 (0.00029)**	**0.008 (0.0073)**	<0.001 (0.25)	**0.007 (0.010)**
0.05	0.001 (0.22)	**0.007 (0.010)**	<0.001 (0.30)	**0.006 (0.015)**	**0.005 (0.034)**	**0.007 (0.011)**
0.1	<0.001 (0.46)	0.003 (0.08)	0.001 (0.15)	**0.005 (0.024)**	0.003 (0.06)	**0.005 (0.027)**
0.2	<0.001 (0.94)	0.003 (0.08)	<0.001 (0.27)	0.002 (0.14)	0.001 (0.22)	**0.005 (0.024)**
0.5	<0.001 (0.80)	0.003 (0.08)	0.003 (0.06)	0.001 (0.23)	0.002 (0.11)	**0.009 (0.0039)**
1	<0.001 (0.66)	0.002 (0.09)	**0.005 (0.034)**	0.001 (0.16)	0.002 (0.09)	**0.010 (0.0035)**
Pickles						
P_T	Childhood IQ	EA 2018	EA 2016	CP	*g*	Intelligence
0.01	<0.001 (0.45)	<0.001 (0.32)	0.001 (0.23)	<0.001 (0.76)	<0.001 (0.66)	<0.001 (0.41)
0.05	<0.001 (0.30)	0.001 (0.17)	0.003 (0.08)	<0.001 (0.77)	0.001 (0.23)	<0.001 (0.44)
0.1	<0.001 (0.25)	<0.001 (0.54)	0.002 (0.11)	<0.001 (0.45)	0.003 (0.06)	<0.001 (0.66)
0.2	<0.001 (0.63)	<0.001 (0.56)	<0.001 (0.55)	<0.001 (0.49)	0.002 (0.15)	<0.001 (0.65)
0.5	<0.001 (0.82)	<0.001 (0.81)	<0.001 (0.93)	<0.001 (0.64)	0.001 (0.20)	<0.001 (0.30)
1	<0.001 (0.87)	<0.001 (0.73)	<0.001 (0.87)	<0.001 (0.78)	0.001 (0.15)	0.001 (0.20)
Meats						
P_T	Childhood IQ	EA 2018	EA 2016	CP	*g*	Intelligence
0.01	<0.001 (0.77)	<0.001 (0.52)	<0.001 (0.83)	<0.001 (0.68)	**0.008 (0.010)**	**0.004 (0.044)**
0.05	0.002 (0.14)	<0.001 (0.36)	<0.001 (0.44)	<0.001 (0.96)	**0.005 (0.029)**	<0.001 (0.56)
0.1	0.001 (0.17)	<0.001 (0.31)	0.001 (0.17)	<0.001 (0.48)	**0.006 (0.024)**	<0.001 (0.91)
0.2	<0.001 (0.42)	<0.001 (0.31)	0.001 (0.17)	0.001 (0.21)	0.001 (0.16)	<0.001 (0.81)
0.5	<0.001 (0.91)	<0.001 (0.33)	0.001 (0.20)	<0.001 (0.81)	<0.001 (0.26)	<0.001 (0.98)
1	<0.001 (0.86)	<0.001 (0.21)	0.001 (0.22)	<0.001 (0.92)	<0.001 (0.29)	<0.001 (0.89)
Soybeans						
P_T	Childhood IQ	EA 2018	EA 2016	CP	*g*	Intelligence
0.01	**0.004 (0.046)**	<0.001 (0.49)	0.001 (0.23)	<0.001 (0.83)	<0.001 (0.76)	0.002 (0.12)
0.05	0.003 (0.08)	<0.001 (0.28)	<0.001 (0.42)	<0.001 (0.92)	<0.001 (0.96)	<0.001 (0.89)
0.1	<0.001 (0.24)	0.002 (0.14)	<0.001 (0.96)	<0.001 (0.26)	<0.001 (0.87)	<0.001 (0.66)
0.2	**0.004 (0.043)**	0.002 (0.12)	<0.001 (0.44)	<0.001 (0.41)	<0.001 (0.79)	<0.001 (0.80)
0.5	**0.004 (0.037)**	<0.001 (0.51)	<0.001 (0.93)	<0.001 (0.35)	<0.001 (0.63)	<0.001 (0.54)
1	0.003 (0.06)	<0.001 (0.52)	<0.001 (0.72)	0.001 (0.19)	<0.001 (0.59)	<0.001 (0.34)

The adjusted R^2 values (corresponding *p*-values) from the regression analyses presented. *P*-values <0.05 are shown in boldface.

Supplementary Table 4. Effects of polygenic scores related to educational attainment (EA) and cognitive functions on fruit consumption in regression analyses adjusted for current disease status (cancer, hypertension, or diabetes mellitus).

Regression adjusted for current cancer status

Fruits						
P_T	Childhood IQ	EA 2018	EA 2016	CP	*g*	Intelligence
0.01	0.001 (0.24)	**0.005 (0.033)**	**0.014 (0.00078)**	**0.007 (0.012)**	<0.001 (0.28)	**0.006 (0.017)**
0.05	0.001 (0.19)	**0.007 (0.013)**	<0.001 (0.47)	**0.006 (0.017)**	**0.005 (0.037)**	**0.007 (0.016)**
0.1	<0.001 (0.42)	0.003 (0.08)	<0.001 (0.26)	**0.005 (0.027)**	0.004 (0.051)	**0.004 (0.040)**
0.2	<0.001 (0.93)	0.003 (0.09)	<0.001 (0.37)	0.001 (0.17)	0.001 (0.19)	**0.005 (0.0070)**
0.5	<0.001 (0.81)	0.003 (0.07)	0.002 (0.10)	<0.001 (0.24)	0.002 (0.10)	**0.008 (0.0070)**
1	<0.001 (0.64)	0.003 (0.08)	0.003 (0.06)	0.001 (0.17)	0.003 (0.07)	**0.008 (0.0076)**

Regression adjusted for current hypertension status

Fruits						
P_T	Childhood IQ	EA 2018	EA 2016	CP	*g*	Intelligence
0.01	0.001 (0.21)	**0.004 (0.039)**	**0.016 (0.00028)**	**0.007 (0.011)**	0.001 (0.21)	**0.008 (0.010)**
0.05	<0.001 (0.43)	**0.007 (0.013)**	<0.001 (0.34)	**0.007 (0.015)**	**0.005 (0.029)**	**0.008 (0.0085)**
0.1	<0.001 (0.77)	0.002 (0.10)	0.001 (0.19)	**0.006 (0.020)**	**0.004 (0.041)**	**0.005 (0.027)**
0.2	<0.001 (0.87)	0.002 (0.10)	<0.001 (0.39)	0.001 (0.16)	0.002 (0.14)	**0.006 (0.023)**
0.5	<0.001 (0.59)	0.003 (0.09)	0.002 (0.10)	0.001 (0.23)	0.002 (0.09)	**0.009 (0.0054)**
1	0.001 (0.41)	0.003 (0.09)	0.003 (0.06)	0.001 (0.16)	0.003 (0.07)	**0.009 (0.0050)**

Regression adjusted for current diabetes mellitus status

Fruits						
P_T	Childhood IQ	EA 2018	EA 2016	CP	*g*	Intelligence
0.01	<0.001 (0.27)	**0.004 (0.044)**	**0.016 (0.00030)**	**0.008 (0.0070)**	<0.001 (0.31)	**0.007 (0.010)**
0.05	0.001 (0.24)	**0.007 (0.014)**	<0.001 (0.27)	**0.007 (0.014)**	**0.004 (0.040)**	**0.007 (0.013)**
0.1	<0.001 (0.51)	0.002 (0.11)	0.001 (0.20)	**0.006 (0.021)**	0.003 (0.06)	**0.005 (0.032)**
0.2	<0.001 (0.99)	0.002 (0.11)	<0.001 (0.40)	0.002 (0.13)	0.001 (0.23)	**0.005 (0.030)**
0.5	<0.001 (0.67)	0.002 (0.11)	0.002 (0.09)	<0.001 (0.25)	0.002 (0.14)	**0.009 (0.0045)**
1	<0.001 (0.51)	0.002 (0.11)	0.004 (0.06)	0.001 (0.17)	0.002 (0.11)	**0.009 (0.0044)**

Adjusted R^2 values (with corresponding *p*-values) from the regression analyses presented. *P*-values <0.05 are shown in boldface.
